# Supplementary material for: Acetylcholinesterase-like proteins are a major component of reproductive trail mucus in the invasive pest land snail, Theba pisana
Source: PLoS One. 2025 May 21;20(5):e0323380. doi: 10.1371/journal.pone.0323380 (PMC12094768; doi:10.1371/journal.pone.0323380)
Supplement: S1 Fig — (DOCX) [file pone.0323380.s001.docx]

**S1 Fig**.

Contig_51164

MVFHTFARSPANIIVMCDSVNELLCLISSQRKMFPLIFMTTVCLLPNVVAVSVPLSSATLTTTLGQITGIRKVIGANQKIDVFYSIPFAKAPVGELRFKPPQPAKPWTGVRDGTQKPNSCWQSIDTNFGRFPGVEMWNPNTQRSEDCLYINVWRPVCTQQCSNQKPKSIMVWIFGGGFYS

Contig_92397

MWARMVAMLCGWLAVLRQVDSSDDLVNMTCTLGTLTGTVVDDPGKASVHVFYGVPYATPPVGNLRFKAPEAAPSWPGARNATALPSSCWQTIDTSFGRSPGVEMWNPNTNMSEDCLYLNLWVPATPTNKAILVWIYGGGFW

Contig_113119

MTGLVVAQAVNGDLATTLGLVQGSLSEHNGTEVRVFYGIPYAKPPVGALRFKPPEPAASWEGTMNATRLPNSCWQTRDTSYNRAPRVEMWNANTNLSEDCLYLNMWIPNTLGAKAVMVWIYGGGFWGGTSTLDIYNGLILAARNNVIVVSFNYRVGPFGFLYCGTPDCPGNVGLLDQVLALKWVKDNAENIGG

Contig_92052

LTTHGLVQGLTENIPVDNGRQVTVDVYWGIPYAKPPVGDLRFQRPVPADPWDGVLQAKTPPNSCIQVPVSPELGLLPSIPGPFSEDCLYLNLWVPRCWSLGRLTTMFWIHGGGFVMGSSTENLYNGANLAASECVIVVS

Contig_83737

MHTKCTGYTMLLMMPLLLGLAIGAVDNKELSPVRKTSSGNIQGYIHTLKDGQKVERYLGVPYATPPVRYMRFKLPQRPRNWTGVRKAISVSAACIQSHLDQMYIKEHAPDYNGNMSEDCLYLNIYVPRRS

Contig_4929

MKMWVLFGILALLPVLVQSAPEIQFPFGKVQGVDKQAQASQKPYIAFYGIPFAEPPINDLRFQPPQPFVGNGSDVVISSDNFRPACLQAGPYSSKVMDEDCLYLNVFTPPDITKTRKVLFWIHGGGFQFGDASQYVPSQLVTDEDVIVVSIQYRLGIFGFLTSQSGDENNGLRDQILALKWTKDNIQAFGGDQNDITIFGESAGSASVSFLSLSPLAKGLFTKAIMQSGTALTYPVLNRRPSVALNSASDKLGCTDSLFYQFWFRQIKXXXXXXXXXXXXXALVCSTQTIFSCLWLAATFCRGVQILSWLMFLT

Contig_5575

MKMCVLFGILALLPVLVQSAPEIQFPFGKVQGVDKQAQASQKPYIAFYGIPFAEPPINDLRFQPPQPFVGNGSDVVISSDNFRPACLQAWPDSSKVMDEDCLYLNVFTPPDITKTRKVLFWIHGGAFQFGDASQYVPSQLVTDEDVIVVSIQYRLGIFGFLTAQSGDENNGLRDQILALKWTKDNIQAFGGDQNDITIFGESAGSASVSFLSISPLAKGLFTKAIMQSGTALSYWALNRRPSVALNSASDKLGCTDSLSYLSWFRQIKGADKIKCLKNKTVSELLSVSSQPQTSAGLFNTDNIF

Contig_83090

MGAMLHGDVLASRGRLVVVTFNYRLGAFGFLHIPHLGIPGNLGLHDQLLAFKWVRENIGQFGGDPTKVTIQGHSAGGVDVGIHVLSPASTGLFRYAILQSGAPTAYWALLPDPNLGNLSTGPRHHVNDLGCSALGAKESLACMKGLNATILSTKHYNHGGDVFMFAPTVDGFFLDDHPLKILDVLARGQTGNVNTQALL

Contig_8577

MATLVYIHGGSLRSGSSTNPLYNGEYLAARECVIVATFNYRLGALGFLFVDTPGFQANVGLFDVYYAIRAIKNEIPSFSGDRSKITVFGSGAGAFLASAFVISSHLRGEVNSVILQSGSPAMHLLYQSRASAEESAVRLATNLSCNRTNKQAMVNCLRRIDARVLTKAFYRKIDGFRYKMVAVMDGTMIRKDPIDYLKNQEINNVSLLIGTNKDDATESLMKMLPGDFNRNVLVPPVFSTRKINRTLRTLLGMVFKSVYFDYLTSQIPIVYN

Contig_8578

SGSSTNPLYNGEYLAARECVIVATFNYRLGALGFLYVDTPGFQANVGLFDVIYAIQAIKNEIPSFSGDRSKITVFGSGTGAFLASAFVISSHLRGEVNSVILQSGSPAMHLLYQSRASAEESAVRLAADLSCNRTNKQAMVNCLRRIDARLLTEAFYRKIDGFRYRMVAVNDGTIIRKDPIDYLKNQEINNVSLLIGTNKNEVTESLMKLLPGDFNRNVPLPSVFSTRKINRTLRTLLGMVFKSVYFDYLTRQIPIVYN

Contig_4592

MFAMAGRGNLVLLVVALLVPCVVDSQTSSAFIQVHLQRYQGTSRNILANGRQIPVETYWSIPYSDPPVGERRFRSSFLSPTAYSHETKNATVPPKACFQQVDNQTIHSYDDMSEDCLYYNIWRPRCNTTTNMATLVYIHGGSLRSGSSANPLYNGEYLAAKQCVIVASFNYRLGVLGFMPFNELNQSNVGVYDVMLAARTIKRDITYFSGDGDKITIFGSGSGAFLASLFVFSRELPHQNMNSVILQSGSPTMQMMSQSRASAEQSVLILARNLSCDTTNRNSIFHCLKNMDARVLTEAFYRKIDGFRYRMVTVMDDSAIRRDPLDFLNFQEINNVSVMIGTNKDXXXXXXXXXXXXXXXXXXXXXXXK

Contig_60659

MGLTDVTLALQWVKDNVGCFGGDPTSVTLFGGGFGGNVVSLALLSPLTRDLFKNAIIQSGSYDIRFVYRSQQASLTTALKFADRLNCSSENRTAIVQCLRQVDAAQLALQYLLLSSTFQDVMVAVTDGPFLPTDPTTALKTGDFKRCEIMVGYNQDDGTYAISRALVDSFNLRREPPALSDKELDSNLTRLLSTLLKPDFIPGVFSQVRQLYNDILKLKDNNSGVALLI

Contig_26296

MGFLDMVLANRWLRDNLDNFGGSPNRITIFGESAGGISISLFLVSPLTRGLFKNVIIQSGNPFLSVYHQSRESATAAGLELSRRLNCGSNSTAEAVRCLRQVDDSALALTHLLLTTNLDYLMSAVTDGFFLTREPITSFQAGDFQPTNILLGTNKDEGTFVVNFLRPATYNFRSQQPLVNLTDQELQQTLTQLLSLTFKPEFIPGIIATVRDLYNYTSLQQAAPNPTFSLLKSVATDLVFACPARLLATFYAK

Contig_83111

MALQWLKDNAVNMGGDPDKITIFGESAGAVSVGFHMMSPLSMHLFTYAVLMSASPTAHWGIQDSDVALNRTQTLACLLGCRARNMAETIDFLRTISPQAITERHWNFSKYYFDTPFAPVVDGHFLPQHPTDLMEQGKVKNTSIISGVVKDEGTYWLLYGFQEIFGSADKTPMSDSDFVTVVGQVLKPLGPQYGTDLVEELTMQEYFHSVPPWKRTYLDATDDISGDTLFKCPVIAFSQFYSSEVKGQVYMYSFEHRVSTNTWPDWLGVAHGYEIELFFGLPFLE

Contig_76447

MMQLWTNFAKTGNPNYNATQEWPKYTAKFKDHVTIDTRGLTPGRGLRQIQCAFLANITNPSPPTEKSAETTFXSSTRRHTFHAIGQ

Contig_5507

MFSMAGRGNLVLLVAALLVPCVVDSQTTSPTVFIPMEYFQGTRRNILVNGLEIPVETYWGIPYSRPPVGLSRFADTRVYIQGEYVQGMVKNATVPPKACFQKVDNRTIHSYDDMSEDCLYYNIWRPRCNTTNMATLVYIHGGSLRSGSSANPLYNGEYLAAKQCVIVASFNYRLGVLGFLPFDIRGSVSNAGLHDVQIAVNIIRIHSSVFSGSEYKITVFGSGTGAFLASTFLFSSYINGVILQSGSPTMQMLYQSRASAEASAMILATNLSCDTTSKTRILTCLRNTDVRVLTEAFYRTINGFHHRMVAVMDGITRTRDFLRNQGLNNVSLMIGTNKDDATESLMKMLPLDFNRNVLVPPVFSTRKIKRTLSFFLGMLYKPQYMPLLTTLSLILYDYENVNRTENSSLAFLSPLIHDLAYTCPMKLVLDAHAAGLRDAYVYSFNHGPDNTSLPAWVGATHRAELAYIFGNSASFSPREIALSDTMQTMWANFAKYGNPTPDNSTGTQWPPYTKQTSERMLVFKEDTQINVSAVNTWKCWSWNAIPKHMKVE

Contig_7063

MAVGMIRITMQGFSGSRKKITVFGSGTGAFLASAFVFSSYVRGEVNSVILQSGSPSMQMLYQSRASAEESAVILATYLSCDTTSNTRILNCLRNINVRVLTEAFYRTINGFHHRMVAVMDGITITRDPLDFLKNQGLNNVSLMIGTNKDDATESLMKMLPLDFNRNVLVPPVFSTQKIKGTLRFFLGLLYKPQYMPLLTTLSLILYDYENVNRTENSSLAFLSPLTHDLAYTCPMKLVLDAHAAVLRDAYVYSFNHGPDNTSLPAWVGATHRAELAYIFGNSASFSPREIALSDTMQTMWANFAKYGNPTPDNSTGTQWPPYTKQTSERMLVFKEDTQINVSAVNTWKCWS

Contig_7249

MAVRRIRLSMIVFSGSSNKITVFGSGTGAFLASAFVFSSHLRGEVNSVILQSGSPTMQMLYQSRASAEASAMILATNLSCDTTSKTRILNCLRNIDVRVLTEAFYRTINGFQHRMVAVMDGITIKRDPLDFLKNQEINNVSVMIGTNKDDATESLMKMLPGDFNRNVLVPPVFSTRKINRTLRTFLGMLYKPQYMPLLTTLSLILYDYENLNRTENSSLAFLSPLTHDLAYTCPMKLVLDAHAAVPRDAYVYSFNHGPDNTSLPAWVGATHRAELAYIFGNTASFSPREIALSDAMQRMWANFAKYGNPTPDNSIGIQWPRYTKQTSERMLVFKEDTQINVSASNTWKCWS

Contig_5509

MFSMAGRGNLVLLVAALLVPCVVDSQTSTVIVAGSYGRFHGTSRNILVNDRQIPVETYWGIPYAQPPIGKRRFKLPKIIRTEPSTIYNAKVPPKACLQKVDNETIHSCEDMSEDCLYYNIWRPRCNTTGMATLVYIHGGSLRSGSSANPLYNGEYLAAKQCVIVASFNYRLGALGFLYVDTPKFRRNAGLFDVIYAMRQIENEIPAFSGDRKKITIFGSGAGAFLASAVFVISPYMRNIVNSVILQSGSPTLQTLYQSRASAEESAMRLAGDLSCNTTSAKSMHLCLKNMDARVLTKAFYRKIDGFRYRMVAVNDGTMIRKDPIEYLKNQEISNVSVMIGTNKDEATESLMKILPRVFNRSVLQPPILSRQKINRTLRTLLGLSYKRDYMNYLTRQVFTIYNYENVNRADNSSLAFLSHVTRDIAYTCPMRLLLDAYAVVPRDVYVYSFNHAPDNTSLPAWVGATHRAELAYIFGNSTSFSPREIALSDTMQRLWANFAKYGNPTPKNSTRTPWPRYTKQTSERMLVFKRDTHVKVAAVNTGKCYFWNAMPSYMKVE

Contig_21801

MVNCLRQVDAHVLMQSFARTIDGDRYRMVTVIDGDIITRSPFDSLRNGHINNVSLMINTNKYKATEAAKTLLLQHSNPHDPLPMLFSDNKINSTLTVFLRKLYNPTHLNYVTTMVKFLYDYEKIKNAGNSSLAFLSPVVQDVVYTCPMRLVLDAYAVAPREVYVYSFNHAPDNSSLTAWGSSTHIADLPYVYGNNIGFSHREYVLNANIKKLLKNFAEYGNPTAGEQVGVDWQRYLQDDEQMLVITEDAQLINGTVQGQRCSFWNTMPAEMKLFF

Contig_117373

MPERFPDGRFKELPESIRNHYKNVYKHKELIYFVYKPWSDPENVTANYMGLSDFIGDVTFVAPTVQTADLLTRSNRTQVYLYSFEHRSELSLYPQWMGVPHGDDL
