# Supplementary figures and images for: Acetylcholinesterase-like proteins are a major component of reproductive trail mucus in the invasive pest land snail, Theba pisana
Source: PLoS One. 2025 May 21;20(5):e0323380. doi: 10.1371/journal.pone.0323380 (PMC12094768; doi:10.1371/journal.pone.0323380)

**S2 Fig.**


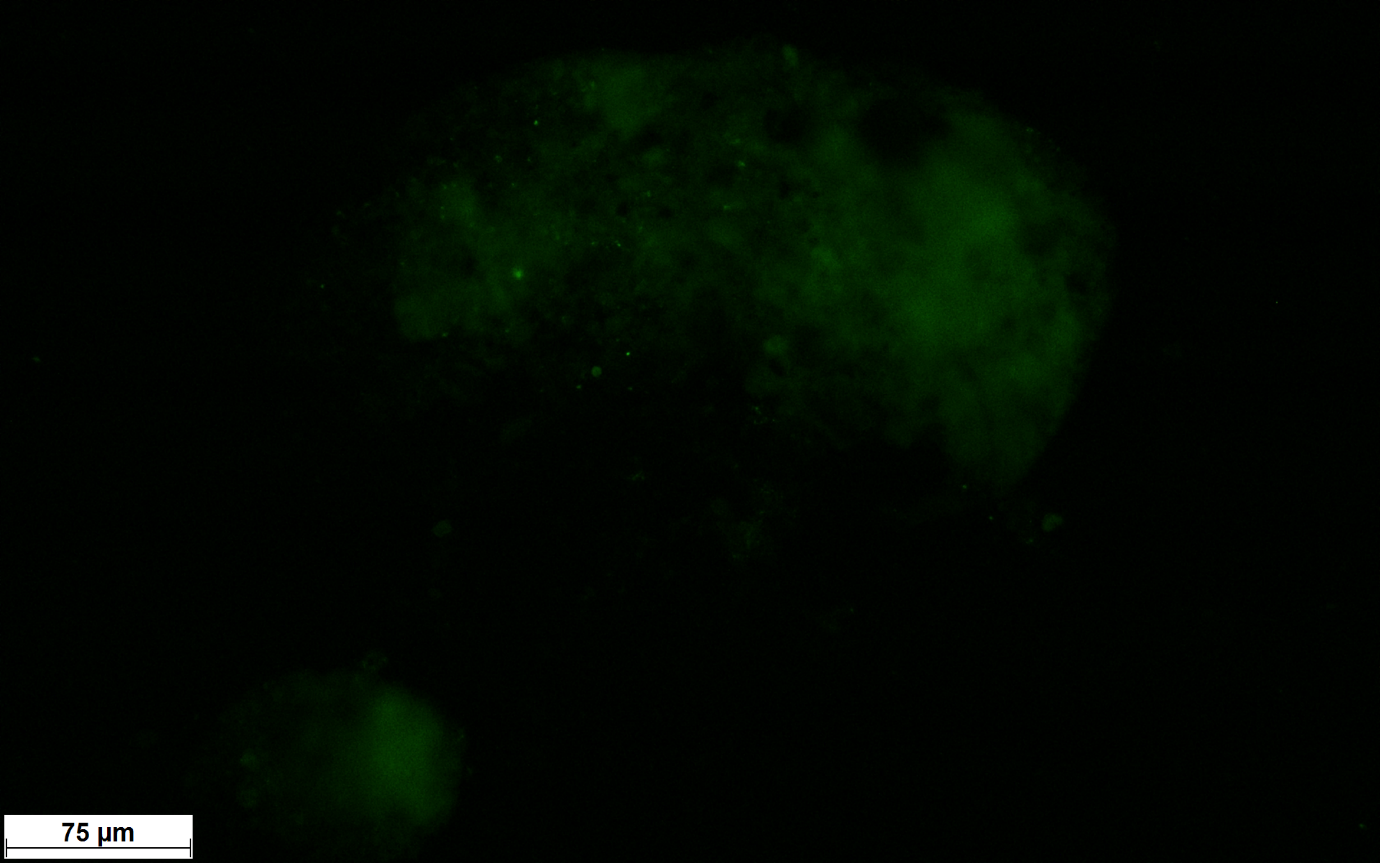

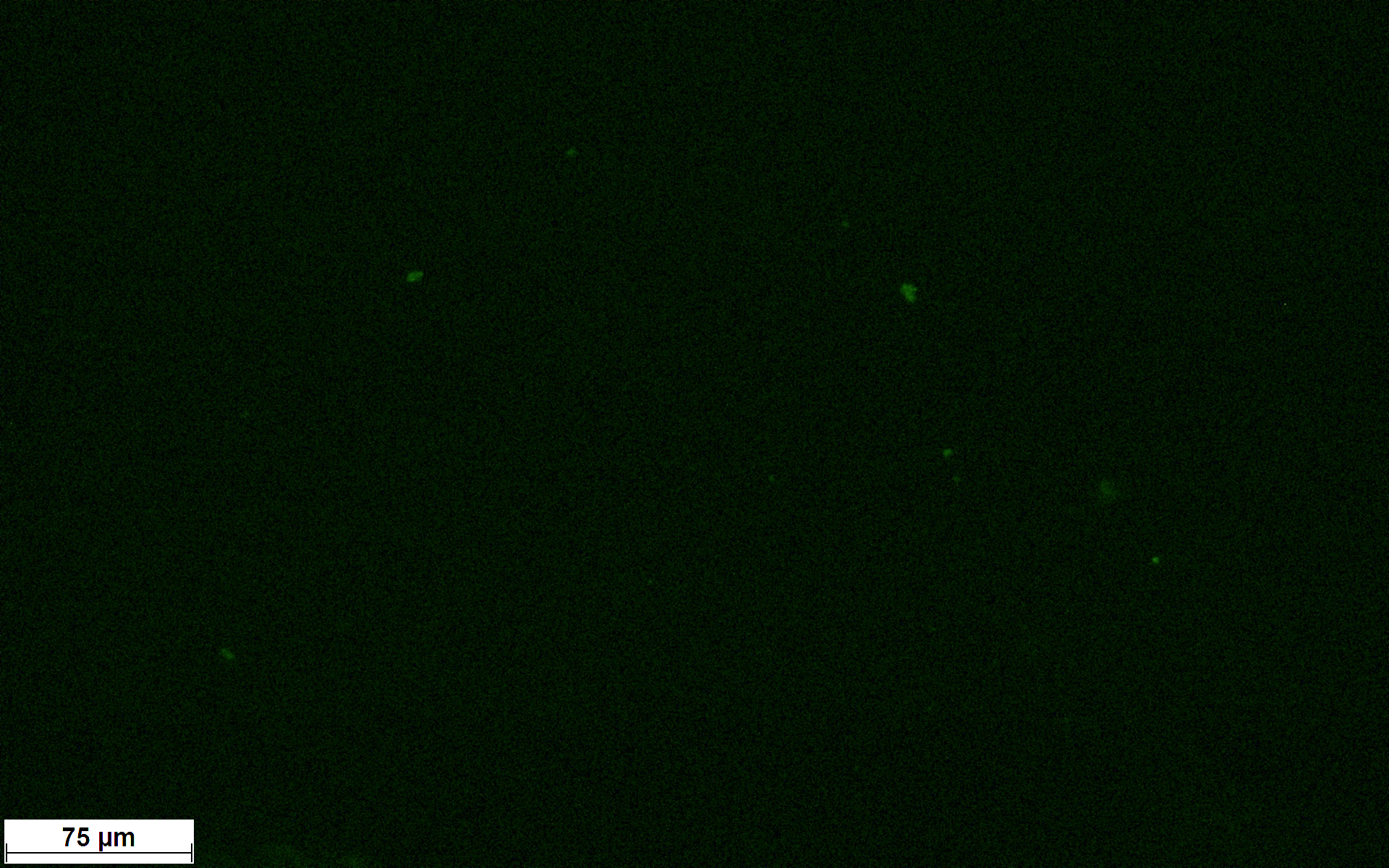


**A**

**B**

Supplement: S2 Fig — (A, B) Immunolocalization showing low/no acetylcholinesterase-like protein (green) in trail mucus. (DOCX) [file pone.0323380.s002.docx]

**S1 Table.**


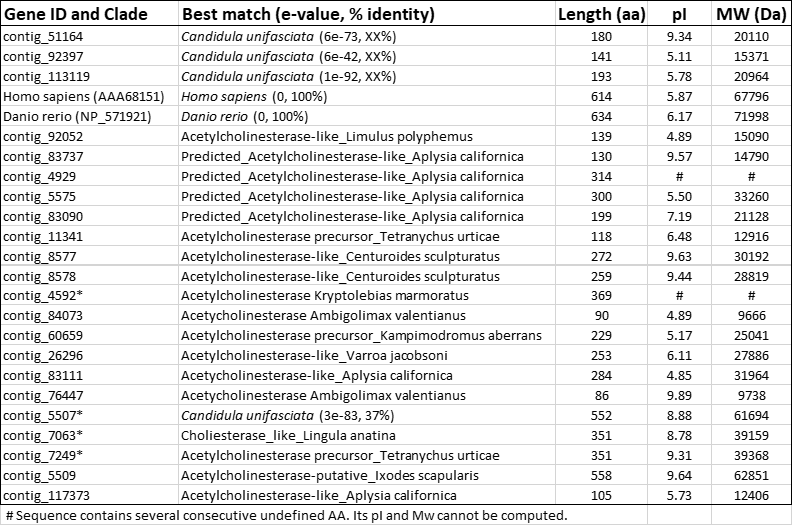

Supplement: S1 Table — (DOCX) [file pone.0323380.s003.docx]
