# Supplementary material for: Acetylcholinesterase-like proteins are a major component of reproductive trail mucus in the invasive pest land snail, Theba pisana
Source: PLoS One. 2025 May 21;20(5):e0323380. doi: 10.1371/journal.pone.0323380 (PMC12094768; doi:10.1371/journal.pone.0323380)
Supplement: S2 Table — Expression is in transcripts per million. R, reproductive stage; NR, non-reproductive stage. (DOCX) [file pone.0323380.s004.docx]

**S2 Table.**

|  | **Mucous gland** | | | | | |
| --- | --- | --- | --- | --- | --- | --- |
| **Gene ID** | **R1** | **R2** | **R3** | **NR1** | **NR2** | **NR3** |
| Contig_7063 | 1400.51 | 1828.85 | 1330.62 | 0 | 0 | 0.15 |
| Contig_5507 | 4507.47 | 6923.03 | 5525.58 | 0.37 | 0.72 | 0.4 |
| Contig_7249 | 1090.16 | 1907.91 | 1571.97 | 0.17 | 0 | 0 |
| Contig_4592 | 2371.08 | 3014.61 | 2850.32 | 0.45 | 0 | 0.74 |
|  | **Albumen gland** | | | | | |
| **Gene** | **R1** | **R2** | **R3** | **NR1** | **NR2** | **NR3** |
| Contig_7063 | 0 | 5.23 | 0 | 0 | 0 | 0 |
| Contig_5507 | 0 | 11.32 | 0.06 | 0 | 0 | 0 |
| Contig_7249 | 0.26 | 2.07 | 0 | 0 | 0 | 0 |
| Contig_4592 | 0 | 7.43 | 0 | 0 | 0.34 | 0.09 |
|  | **Foot** | | | | | |
| **Gene** | **R1** | **R2** | **R3** | **NR1** | **NR2** | **NR3** |
| Contig_7063 | 0.59 | 0.83 | 0.13 | 0 | 0.57 | 0 |
| Contig_5507 | 7.48 | 2.97 | 2.92 | 0.43 | 2.89 | 3.18 |
| Contig_7249 | 0.44 | 0 | 0.38 | 0 | 0 | 0 |
| Contig_4592 | 3.84 | 2.13 | 2.71 | 1.43 | 2.17 | 0.54 |
|  | **Cerebral ganglia** | | | | | |
| **Gene** | **R1** | **R2** | **R3** | **NR1** | **NR2** | **NR3** |
| Contig_7063 | 0.33 | 0.8 | 0.79 | 0 | 0.39 | 0.15 |
| Contig_5507 | 0.35 | 0.52 | 0.59 | 0.3 | 0.14 | 0.47 |
| Contig_7249 | 0.16 | 0.64 | 0.16 | 0 | 0.13 | 0 |
| Contig_4592 | 0.8 | 1.23 | 0.88 | 0.43 | 0.39 | 0.15 |
