# Supplementary material for: Acetylcholinesterase-like proteins are a major component of reproductive trail mucus in the invasive pest land snail, Theba pisana
Source: PLoS One. 2025 May 21;20(5):e0323380. doi: 10.1371/journal.pone.0323380 (PMC12094768; doi:10.1371/journal.pone.0323380)
Supplement: Attachment — Submitted filename: Responses to reviewer 1 and 2.docx [file pone.0323380.s006.docx]

**Reviewer #1:**

**Specific comments:**

**1. The author needs to provide the reference for following the specific protocol for the Identification and characterisation of acetylcholinesterase-like genes (Line 141-160).**

RESPONSE: A reference has now been included to the Methods that justifies the protocol used – i.e. The general protocol used to identify and characterise snail genes was as described by Ballard et al., 2021 and Ballard et al., 2025.

**2. Italicize the word In Vitro (Line 367).**

RESPONSE: *In vitro* has now been italicized.

**3. Kindly add full stop before the beginning of a new sentence i.e., For pesticides in the Line 364.**

RESPONSE: A full stop has been added.

**4. It’s advisable to authors to strictly follow one font style throughout the manuscript.**

RESPONSE: We agree and have ensure consistency in font style throughout. i.e. Times New Roman, font size 12.

**5. Provide single space before the ‘a notorious garden pest’ (line 378).**

RESPONSE: A single space has now been added.

**6. Locate the epithelial folds and other described things in the Figure 4 probably using arrows.**

RESPONSE: To assist in recognition of the relevant histological features, we have annotated the images to provide labelled spatial location of the mucous gland lumen, smooth muscle and villi that represent the epithelial folds. In addition, the scale bars have been updated for clarity.

**7. Author needs to mention the accession number of AChE-like proteins genes in Theba pisana, in the Figure 1A and in the text also.**

RESPONSE: We now include in the main text (as **S1 File** and **S2 File**) and Figure 1 legend (as **S1 File)** where all AChE-like sequences used in the analyses can be found. The NCBI BioProject database under accession number PRJNA858108 is provided in the Methods and Resources for those who want to assess the raw data.

**8. Kindly go through the manuscript for proof reading again to avoid text mistakes.**

RESPONSE: All authors have proofread the manuscript prior to our resubmission.

**9. The following edits have been made as suggested:**

Line 250 – edited Microscope images were taken using an upright Leica fluorescence microscope with a CCD camera, to ‘Images were taken using an upright Leica fluorescence microscope with a CCD camera.’

Line 256– edited the 4% paraformaldehyde was pipetted onto each slide… to ‘Paraformaldehyde (4%) was pipetted onto each slide’…

Line 271 – edited the majority of AChE-like proteins were relatively... to ‘Most AChE-like proteins were’...

Line 302 – edited Initially, specificity was tested using western blot… to ‘Initially, the specificity was tested using western blot’…

Line 386 – edited 4 AChE-like proteins were comparative identified… to ‘4 AChE-like proteins were identified’…

Line 401 – edited which shows the versatility of secretory AChE function to ‘which shows the versatility of secretory AChE functions…’

Line 419 – edited communities of land and water dwelling snail pests, however, to ‘communities of land and water dwelling snail pests, yet…’

Line 431 – edited and ultimately get spread into the… to ‘and ultimately spread into the’ …

Line 436 to 445– edited This would assist in determining its ability to function similarly to its natural neuronal role, or purely to scavenge organophosphates. It is clear that *T. pisana* has diversified the number and location of AChE-like proteins, compared to vertebrates, but it would be of interest of observe… to

‘This would assist in determining whether the function of AChE-like proteins in land snails includes both a neuronal role along with the ability to scavenge organophosphates. *Theba pisana* has diversified the number and location of AChE-like proteins, compared to vertebrates. It would be of interest to observe’…

**9. What is the hypothesis behind this study?**

RESPONSE: We thank the reviewer for recognising no clear hypothesis statement. In the Introduction section, we have added the study hypothesis - We hypothesized that land snails secrete AChE proteins into their trail mucus, which may function to neutralize pesticides.

**10. Have authors looked for the possibility of other genes other than acetylcholinesterase-like genes in the trail mucous of snails and on the idea that other genes might be involved in the resistance against the pesticides?**

RESPONSE: We agree, and following the recently published manuscript in Biology - <https://www.mdpi.com/2079-7737/14/3/294> - where the trail mucus of a related land snail species (Cernuella virgata) was investigated, we have added to the discussion information that describes multiple different proteins that are temporally present in adult *C. virgata* mucus. We postulate that there are other proteins that could help facilitate pesticide resistance and should be further investigated.

**Reviewer #2:**

1- **Please explain more details about Acetylcholinesterase analysis in material and method**

RESPONSE: To help clarify details of the AChE identification and analysis, we have added new information to the Material and Method section:

- Line 143 – The general protocol used to identify and characterise snail genes was as described by Ballard et al., 2021 (1, 2)
- Line 165 – Trail mucus was collected using a method previously described by Ballard et al., 2021(1, 2)

2- **Please improve the English style**

RESPONSE: All authors have proofread the manuscript prior to our resubmission. Changes include those listed below, with line numbers based on original submission -

Line 13 – edited yet are often broad-spectrum and species develop resistance… to ‘yet are often broad-spectrum, leading to the development of resistance in target species’…

Line 19 – edited clades; one clade included… to ‘clades, with clade including’…

Line 52 – edited (e.g., wheat, barley, oat), which can clog farming machinery… to ‘(e.g., wheat, barley, oat); as a result, snails can be inadvertently harvested which can clog farming machinery’…

Line 53 – edited Aestivation is a behavioural response to higher temperature that… to ‘Aestivation is a behavioural response to higher temperatures that’…

Line 85 – edited This research investigated a potential role for resistance-modified AChE in invasive pest land snails, using T. pisana. Using tissue- and … to ‘We hypothesized that T. pisana secrete AChE proteins into their trail mucus, which may function to neutralize pesticides. Utilising tissue- and’…

Line 89 – edited This finding helps provide some explanation as to the potential molecular cause of pesticide resistance… to ‘This finding helps to provide some explanation as to the potential of molecular signalling mechanisms involved in pesticide resistance and’…

Line 128 – edited immediately snapped frozen… to ‘immediately snap-frozen’…

Line 166 – edited *T. pisana* were, rinsed… to *T. pisana* were rinsed’…

Line 197 – edited the LC-MS/MS data were imported… to ‘the LC-MS/MS data was imported’…

Line 214 – edited into new tubes, then… to ‘into new tubes and;’…

Line 223 – edited was added and rocked… to ‘was added and the membrane rocked’…

Line 233 – edited samples were further dehydrated to 100% ethanol… to ‘samples were further dehydrated in 100% ethanol’…

Line 242 – edited in 1 x PBS, 0.1% Tween (PBST)… to ‘in 1 x PBS, then 0.1% Tween (PBST)’…

Line 243 – edited added to slides and incubated… to ‘added to the slides which were incubated’…

Line 256 – edited onto each slide and left for 1 h… to ‘onto the slides, covered, and left to sit for 1 h’…

Line 270 – edited addition of (GenBank accession number PRJNA858108)’…

Line 386 - edited 4 AChE-like proteins were comparative identified; comparative RNA-seq… to ‘4 AChE-like proteins were identified; Comparative RNA-seq’…

Line 388 – edited AChE-like proteins had never… to ‘AChE-like proteins have never’…

Line 416 – edited agricultural gains to mitigate invasiveness… to ‘agricultural attempts of control towards mitigation’…

Line 421 – edited could lead to research… to ‘could lead to further research’…

Line 427 – edited this research provided… to ‘this research provides’…

Line 432 – edited likely mechanism to avoid the direct impact… to ‘likely mechanism to protect the snail from the direct impact’…

Line 433 – edited speculated as a mechanism… to ‘speculated to be a mechanism’…

Line 434 – edited first description of a land snail… to ‘first description of this function in a land snail’…

**3- What is the effect of Acetylcholinesterase in the life cycle of pest land snail**

RESPONSE: In the discussion section, we have added information regarding the recognised effect of acetylcholinesterase on the life cycle of the snail, and the molluscs in general:

There exists a vast amount of information surrounding vertebrate AChE due to its significant role in terminating acetylcholinesterase-mediated neurotransmission (3-6). In molluscs, this role has been confirmed through studies in *Aplysia* (7) and *Mytilus* (8). However, AChE potential role in overcoming OPs has been more intensively studied in invertebrates, particularly in the fruit fly (*Drosophila*) (9) and ticks (*Rhipicephalus*) (10). Molluscan gastropods are also excellent invertebrate models for investigating harmful biomarkers in an environment, especially since AChE activity functions in the space of inhibition against certain pesticides (11).

**References**

1.	Ballard KR, Klein AH, Hayes RA, Wang T, Cummins SF. The protein and volatile components of trail mucus in the Common Garden Snail, Cornu aspersum. PloS one. 2021;16(5):e0251565-e.

2.	Ballard KR, Ventura T, Wang T, Elizur A, Cummins SF. Mucus Trail Proteins May Infer Reproductive Readiness for Land Snails. Biology. 2025;14(3):294.

3.	Herz F, Kaplan E. A review: human erythrocyte acetylcholinesterase. Pediatric Research. 1973;7(4):204-14.

4.	Patočka J, Kuča K, Jun D. Acetylcholinesterase and butyrylcholinesterase–important enzymes of human body. Acta Medica (Hradec Kralove). 2004;47(4):215-28.

5.	Saldanha C. Human erythrocyte acetylcholinesterase in health and disease. Molecules. 2017;22(9):1499.

6.	Thomsen T, Kaden B, Fischer J, Bickel U, Barz H, Gusztony G, et al. Inhibition of acetylcholinesterase activity in human brain tissue and erythrocytes by galanthamine, physostigmine and tacrine. 1991.

7.	Srivatsan M, Peretz B. Acetylcholinesterase promotes regeneration of neurites in cultured adult neurons of Aplysia. Neuroscience. 1997;77(3):921-31.

8.	Santos GPCd, Assis CRDd, Oliveira VM, Cahu TB, Silva VL, Santos JF, et al. Acetylcholinesterase from the charru mussel Mytella charruana: kinetic characterization, physicochemical properties and potential as in vitro biomarker in environmental monitoring of mollusk extraction areas. Comparative Biochemistry and Physiology Part C: Toxicology & Pharmacology. 2022;252:109225.

9.	Fournier D, Mutero A, Pralavorio M, Bride J-M. Drosophila acetylcholinesterase: Mechanisms of resistance to organophosphates. Chemico-Biological Interactions. 1993;87(1):233-8.

10.	Singh H, Nandi A, Singh NK. STATUS OF ACETYLCHOLINESTERASE MEDIATED ORGANOPHOSPHATE RESISTANCE IN CATTLE TICK, RHIPICEPHALUS MICROPLUS (ACARI: IXODIDAE). Exploratory Animal & Medical Research. 2024;14.

11.	da Silva Lucas RM, Gonçalves FJ, Rodrigues FA, de Oliveira LCC. Acetylcholinesterase Activity Evaluated in Molluscs–A Bibliometric Analysis. Brazilian Journal of Development. 2025;11(1):e76398-e.
